# *Rhadinorhynchus oligospinosus* n. sp. (Acanthocephala, Rhadinorhynchidae) from mackerels in the Pacific Ocean off Peru and related rhadinorhynchids in the Pacific, with notes on metal analysis

**DOI:** 10.1051/parasite/2017022

**Published:** 2017-06-08

**Authors:** Omar M. Amin, Richard A. Heckmann

**Affiliations:** 1 Institute of Parasitic Diseases 11445 E. Via Linda # 2-419 Scottsdale AZ 85259 USA; 2 Department of Biology, Brigham Young University Provo UT 84602 USA

**Keywords:** Acanthocephala, *Rhadinorhynchus oligospinosus* n. sp., Peru, Pacific mackerels, Description, EDAX analysis

## Abstract

Specimens of a new species of *Rhadinorhynchus* Lühe, 1911 are described from the chub mackerel *Scomber japonicus* (Scombridae) and the Chilean Jack mackerel *Trachurus murphyi* (Carangidae) (possibly a subspecies of *Trachurus symmetricus*) from the Pacific Ocean off the Peruvian coast at the Port of Chicama, La Libertad. Specimens of *Rhadinorhynchus oligospinosus* n. sp. are somewhat small having 11–14 rows of alternating proboscis hooks with 20–22 hooks each with posteriormost hooks in a continuous ring. Ventral hooks are robust with prominent roots but dorsal hooks are slender and shorter with discoid roots. Trunk spines are in two zones separated by a non-spiny region. Anterior trunk spines are in 2–3 complete circles but posterior spines are only ventral and lateral, and do not extend posterior to the level of the posterior end of the proboscis receptacle in both sexes. The new species is closest to *Rhadinorhynchus seriolae* (Yamaguti, 1963) Golvan, 1969 found in Japanese and Australian waters, but not as close to 19 other species found in the same Pacific waters off Australia, Japan, and Vietnam. In *R. seriolae*, posterior trunk spines extend well past the receptacle in females, among other diagnostic differences. Proboscis hooks of the new species were analyzed for chemical elements using X-ray in conjunction with EDAX (energy-dispersive analysis for X-ray) software; sulfur had a higher concentration at the edge than the middle of cut hooks.

## Abstract (fr)

Des spécimens d’une nouvelle espèce de *Rhadinorhynchus* Lühe, 1911 sont décrits chez le maquereau *Scomber japonicus* (Scombridae) et le Chinchard du Chili *Trachurus murphyi* (Carangidae) (éventuellement une sous-espèce de *Trachurus symmetricus*) de l’océan Pacifique au large de la côte du Pérou, Port de Chicama, La Libertad. Les spécimens de *Rhadinorhynchus oligospinosus* n. sp. sont assez petits, avec 11-14 rangées de crochets de proboscis alternés avec 20-22 crochets chacune avec des crochets postérieurs dans un anneau continu. Les crochets ventraux sont robustes avec des racines importantes, mais les crochets dorsaux sont minces et plus courts avec des racines discoïdes. Les épines du tronc sont dans deux zones séparées par une région non épineuse. Les épines du tronc antérieur sont en 2-3 cercles complets, mais les épines postérieures ne sont que ventrales et latérales et ne s’étendent pas plus en arrière que le niveau de l’extrémité postérieure du réceptacle de proboscis chez les deux sexes. La nouvelle espèce est proche de *Rhadinorhynchus seriolae* (Yamaguti, 1963) Golvan, 1969, trouvée dans les eaux japonaises et australiennes, mais moins proche de 19 autres espèces trouvées dans les eaux du Pacifique au large de l’Australie, du Japon et du Vietnam. Chez *R. seriolae*, les épines postérieures du tronc s’étendent bien au-delà du réceptacle chez les femelles, entre autres différences diagnostiques. Les crochets du proboscis de la nouvelle espèce ont été analysés pour des éléments chimiques utilisant des rayons X en conjonction avec le logiciel EDAX (Analyse d’Énergie Dispersive pour Rayons X); le soufre a une concentration plus élevée au bord qu’au milieu des crochets découpés.

## Introduction

An increasing number of research publications on the acanthocephalan parasites of marine fishes in the east Pacific Ocean off the coast of Peru have made their appearance in the past few years. For example, Oliva et al. [29] reported on the ecology of metazoan parasites of *Stellifer minor* (Tschudi), Tantaleán et al. [38] surveyed the acanthocephalans of Peru, and Amin et al. [3] redescribed *Rhadinorhynchus ornatus* Van Cleave, 1918 from skipjack tuna, *Katsuwonus pelamis* (Linn.) with special reference to the presence of microtriches on its trunk, Cruces et al. [8] reported on the parasites of the chub mackerel, *Scomber japonicus* Houttuyn off Peru, and Minaya et al. [24] discussed the community of parasites of the Peruvian weakfish, *Cynoscion analis* (Jenyns) from the Peruvian Pacific. Additional reports of parasites of *S. japonicus* from the Pacific Ocean off the central zone of Peru include those of Luque and Iannacone [23], Rivera et al. [32], Iannacone and Luque [17], Llerena et al. [22], Oliva et al. [29], Cabrera and Tantaleán [6], and Ruelas and Cordova [33–35].

Elsewhere in Pacific South America, Oliva et al. [30] studied the metazoan parasites of *S. japonicus* of the Atlantic Ocean (Brazil) and the Pacific Ocean (Chile), and Munoz and Olmos [25, 26] revised the parasitic fauna of *S. japonicus* from Chile. The parasitic fauna of *Trachurus murphyi* Nichols or *Trachurus symmetricus murphyi* Nichols is rarely reported; see Oliva [27, 28] who reported no *Rhadinorhynchus* in Peruvian or Chilean waters and George-Nascimento [12] who reported *Rhadinorhynchus trachuri* Harada, 1935 off Chile.

Our studies from *S. japonicus* and the Chilean Jack Mackerel *Trachurus murphyi* Nichols in the Peruvian Pacific revealed the presence of a new species of *Rhadinorhynchus*. The latter fish species is considered a subspecies of *Trachurus symmetricus* Ayres by many [9, 10, 31]. Specimens of the same species have been misidentified as *Rhadinorhynchus pristis* (Rudolphi, 1802) in other reports from Peru in other collections [8]. “*Rhadinorhynchus pristis”* was not found in *S. japonicus* from Callao, Peru or from Antofagasta, Chile but was reported from *S. japonicus* in Rio de Janeiro, Brazil and Madeira, Portugal [30]. Our description adds a new taxon to the acanthocephalan fauna of the Pacific Ocean in South America. Re-examination of the Acanthocephala of *S. japonicus* and *T. murphyi* with the aim of correcting previous records would be highly desirable.

## Materials and methods

### Collections

Thirty-one specimens of *S. japonicus* were obtained from the local fish market of the Port of Chicama, Ascope Province, La Libertad, Peru (07°42′S, 79°37′W) in March, 2014. The specimens were immediately fixed in 5% formalin and transferred to the Faculty of Biological Sciences, Ricardo Palma University, Santiago de Surco, Lima, Peru for dissection and recovery of parasites. In all, 12 male and 19 female hosts measured 30.7–38.6 cm (mean of 34.2 cm) and weighed 487–516 g (495 g). Findings on 12 species of external and internal helminth parasites, including a species of *Rhadinorhynchus* conspecific with our new species misidentified as *R. pristis*, were also reported from the same collection by Cruces et al. [8]. Additional specimens of the same species of *Rhadinorhynchus* were also collected from *S. japonicus* and from the Chilean Jack Mackerel *Trachurus murphyi* by Biologa Asucena Naupay, Facultad de Ciencias Biológicas, Universidad Nacional Mayor de San Marcos. They were initially fixed in 70% ethanol and 5% formalin and made available to us. A total of 21 specimens were transferred to our Arizona, USA laboratory for processing and further studies. Six specimens were used for scanning electron microscopy (SEM) studies, 3 specimens were saved for future DNA analysis, and 12 specimens (8 males, 4 females) were processed for optical microscopy as follows.

### Study of Acanthocephala

Worms were punctured with a fine needle and subsequently stained in Mayer’s acid carmine, destained in 4% hydrochloric acid in 70% ethanol, dehydrated in ascending concentrations of ethanol (24 h each), and cleared in 100% xylene then in 50% Canada balsam and 50% xylene (24 h each). Whole worms were then mounted in Canada balsam. Measurements are in micrometers, unless otherwise noted; the range is followed by the mean values between parentheses. Width measurements represent maximum width. Trunk length does not include proboscis, neck, or bursa. Line drawings were created by using a Ken-A-Vision micro-projector (Ward’s Biological Supply Co., Rochester, NY), which uses cool quartz iodine 150 W illumination. Color-coded objectives, 10×, 20×, and 43× lenses, are used. Images of stained whole mounted specimens were projected vertically on 300 series Bristol draft paper (Strathmore, Westfield, MA), then traced and inked with India ink. Projected images were identical to the actual specimens being projected. The completed line drawings were subsequently scanned at 600 pixels on a USB and then downloaded to a computer.

Type specimens were deposited in the University of Nebraska’s State Museum’s Harold W. Manter Laboratory of Parasitology (HWML) collection in Lincoln, NE, USA.

### SEM (scanning electron microscopy)

Samples of parasite that had been fixed and stored in 70% ethanol were processed following standard methods [21]. These included critical point drying (CPD) in sample baskets and mounting on SEM sample mounts (stubs) using conductive double-sided carbon tape. Samples were coated with gold and palladium for 3 min using a Polaron #3500 sputter coater (Quorum (Q150 TES) www.quorumtech.com) establishing an approximate thickness of 20 nm. Samples were placed and observed in an FEI Helios 660 NanoLab DualBeam (FEI, Hillsboro, OR) scanning electron microscope, with digital images obtained in the Nanolab software system (FEI, Hillsboro, OR), and then transferred to a USB for future reference. Images were taken at various magnifications. Samples were received under low vacuum conditions using 10 KV, spot size 2, 0.7 Torr using a GSE detector.

### X-ray microanalysis (XEDs), EDAX (energy-dispersive analysis for X-ray)

Standard methods were used for preparation, similar to the SEM procedure. Specimens were examined and positioned with the above SEM instrument, which was equipped with a Phoenix energy-dispersive X-ray analyzer (FEI, Hillsboro, OR). X-ray spot analysis and live scan analysis were performed at 16 KV with a spot size of 5, and results were recorded on charts and stored with digital imaging software attached to a computer. The TEAM *(Texture and Elemental Analytical Microscopy) software system (FEI, Hillsboro, OR) was used. Data were stored on a USB for future analysis. The data included weight, percent, and atom percent of the detected elements, following correction factors.

### Ion sectioning of hooks

A dual-beam SEM with a gallium (Ga) ion source (GIS) was used for the LIMS (liquid ion metal source) part of the process. The hooks of the acanthocephalans were sectioned using a probe current between 0.2 nA and 2.1 nA according to the rate at which the area is cut. The time of cutting was based on the nature and sensitivity of the tissue. Following the initial cut, the sample also went through a milling process to obtain a smooth surface. The cut was then analyzed for chemical ions with an electron beam (Tungsten) to obtain an X-ray spectrum. Results were stored with the attached imaging software then archived for future use. The intensity of the GIS was variable due to the nature of the material being cut.

## Results

### Collections

Twenty-one specimens of a new species of *Rhadinorhynchus* Lühe, 1911 are described from two species of perciform fish, the chub mackerel *S. japonicus* (Scombridae) and the Chilean Jack mackerel *T. murphyi* (Carangidae). *Scomber japonicus* is widespread in the Indo-Pacific and is usually found in the north-western and eastern Pacific within 37 km off the coast in waters between 10 and 22 °C [7]. *Trachurus murphyi* has been identified in the South Pacific off the coasts of Chile, Ecuador, and Peru, and around New Zealand and South Australia [1, 7, 39]. It is considered the southern sister species of the Pacific Jack Mackerel *Trachurus symmetricus* Ayres, which is usually found in pelagic and off-shore environments off the Pacific coast of North America but may extend as far south as the Galapagos Islands where it could become sympatric with *T. murphyi* [8]. *Trachurus murphyi* was considered a subspecies of *T. symmetricus* until Poulin et al. [31] resolved its independent specific status even though [9] and other authors still recognize it as *Trachurus symmetricus murphyi* Nichols. These mackerels feed on copepods and other crustacean-like mysids and euphausiids, as well as on small fish and squids but on zooplankton as juveniles [6, 9, 20]. None of these food items has been recognized to date as an intermediate host for rhadinorhynchid acanthocephalans in the Pacific.

#### *Rhadinorhynchus oligospinosus* n. sp.

urn:lsid:zoobank.org:act:B4F239E4-8570-41BD-BC20-1A1D6C0BB79E

Family: Rhadinorhynchidae Travassos, 1923

Genus: *Rhadinorhynchus* Lühe, 1911

Type host: Chilean Jack mackerel *Trachurus murphyi* Nichols (Carangidae) (also regarded as subspecies of *Trachurus symmetricus* Ayres (Carangidae) by some accounts)

Other host: Chub mackerel, *Scomber japonicus* Houttuyn (Scombridae)

Type locality: The Pacific Ocean off the Peruvian coast at the Port of Chicama, La Libertad, Peru (07°42′S, 79°37′W).

Site of infection: Intestine.

Type specimens: University of Nebraska’s State Museum’s Harold W. Manter Laboratory (HWML) collection in Lincoln, Nebraska, Collection No. 103065 (holotype male and paratypes on one slide) and No. 103066 (allotype female and paratypes on one slide).

Etymology: The name of the new species denotes the posterior extent of the distribution of the few trunk spines up to the level of the posterior end of the receptacle in both males and females.

#### Description (Figs. 1–6)

**Figure 1.**
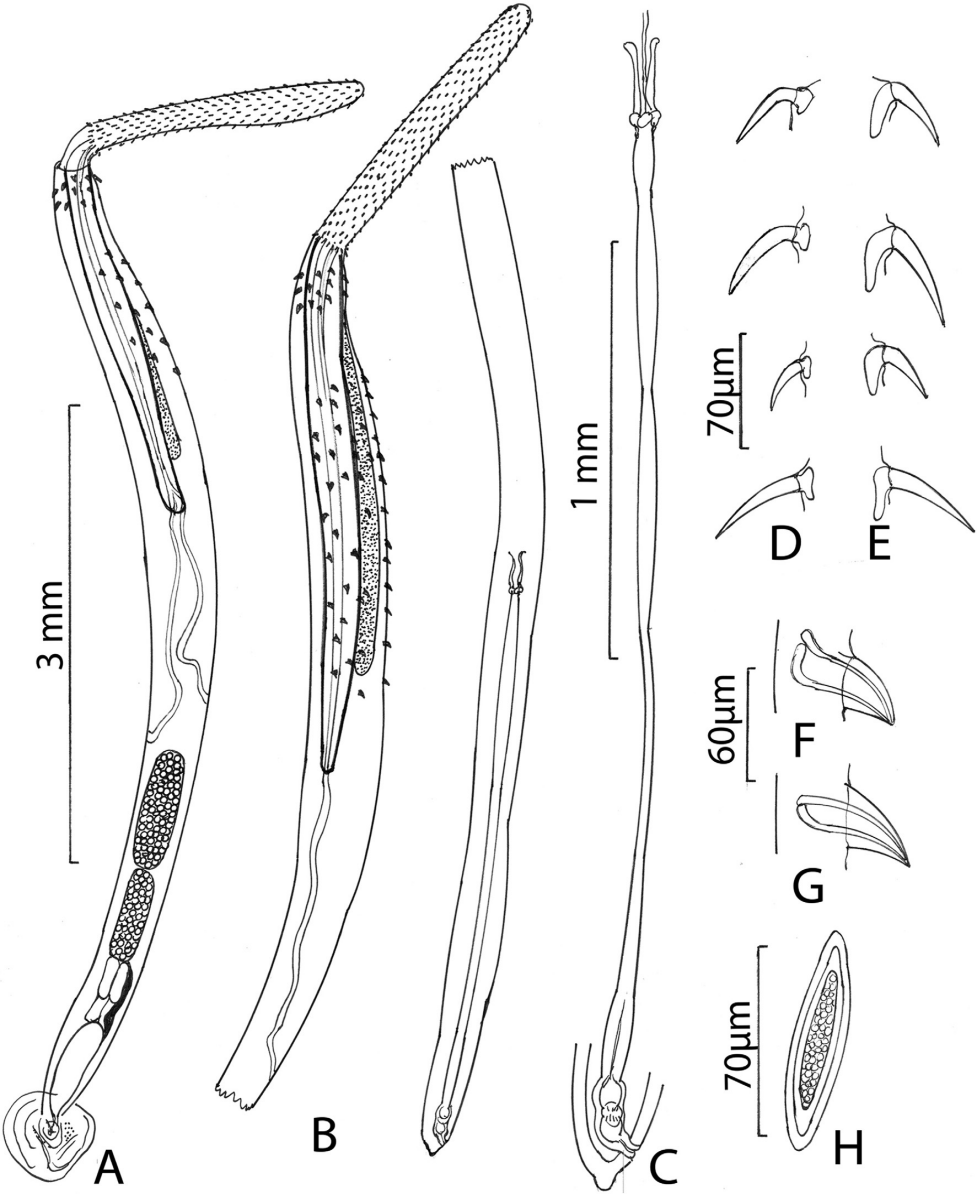
Line drawings of specimens of *Rhadinorhynchus oligospinosus* from the mackerels *Scomber japonicus* and *Trachurus murphyi* from off the Pacific coast of Peru. Normal body wall thickness (A, B) not shown. (A) Holotype male. Note the lemniscus being shorter than the receptacle, the restricted distribution of posterior trunk spines, and posterior position of the reproductive system. The common sperm duct is lateral and ventral to the four cement glands. (B) Allotype female with the same distribution of lemniscus and posterior trunk spines as in the male. Measurement scale is the same as the male (A). (C) Detail of the long female reproductive system of specimens in (B); note the simple uterine bell and glands. (D) Apical, middle, posteriormost, and basal dorsal proboscis hooks in a male specimen. (E) Apical, middle, posteriormost, and basal ventral proboscis hooks in the same male specimen. Note the dorso-ventral diversification of hooks and the reduced dorsal hook roots. (F) A profile of a ventral anterior trunk spine in a female specimen. (G) A profile of a ventral posterior trunk spine in the same female specimen. (H) A ripe egg.

**Figure 2.**
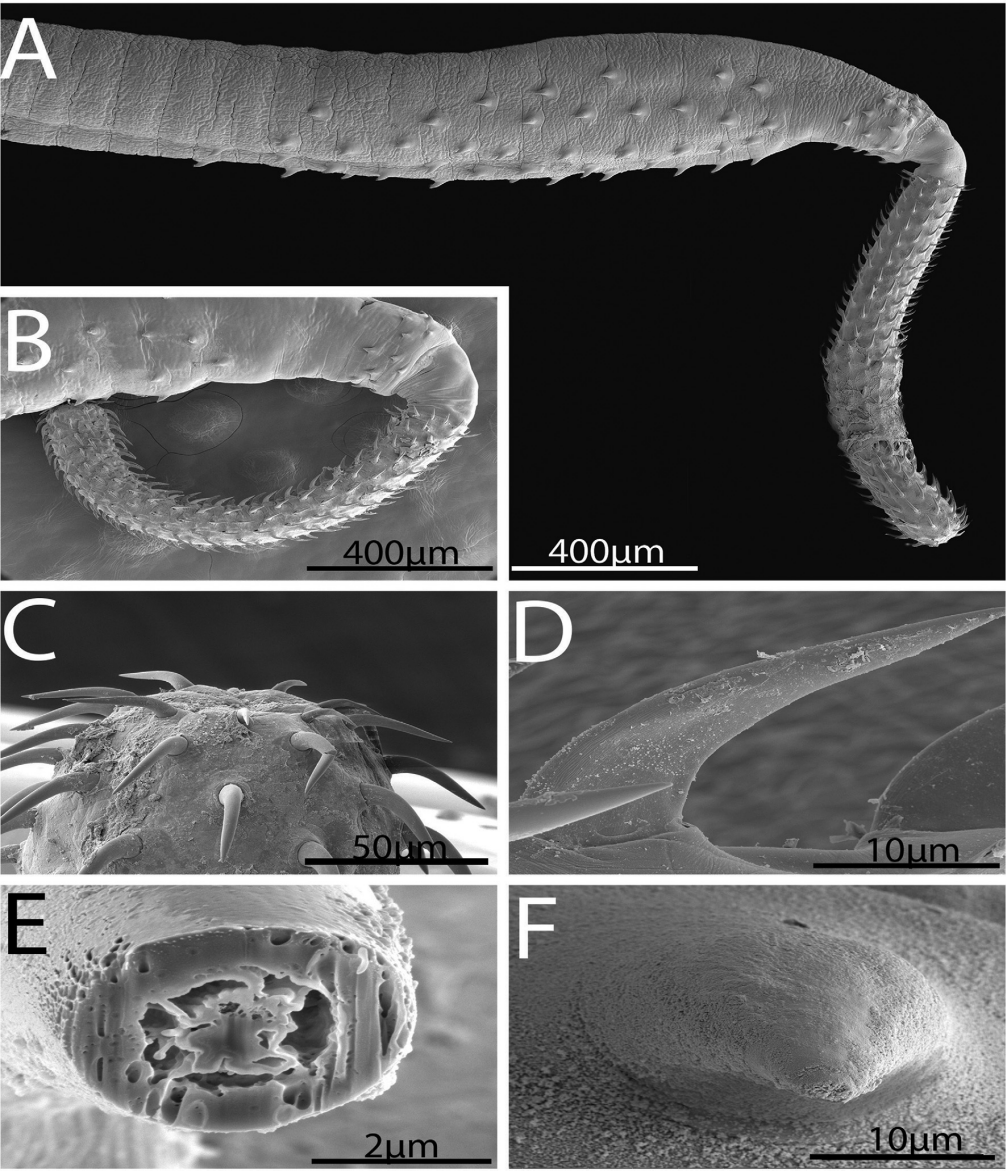
SEM image of specimens of *Rhadinorhynchus oligospinosus* from *Scomber japonicus* and *Trachurus murphyi* from off the Pacific coast of Peru. (A) A profile of the anterior part of a male specimen showing the neck and proboscis curvature and the distribution of trunk spines. (B) The proboscis and curved neck of another specimen. (C) The anterior end of a proboscis showing the minute size of the apical hooks compared to subapical hooks. (D) A typical hook at mid-proboscis showing its curvature and its thickness at base. (E) A gallium cut section of a proboscis hook showing its thick cortical layer and partially vacuolated center. (F) A basal view of a trunk spine showing its broad base.

**Figure 3.**
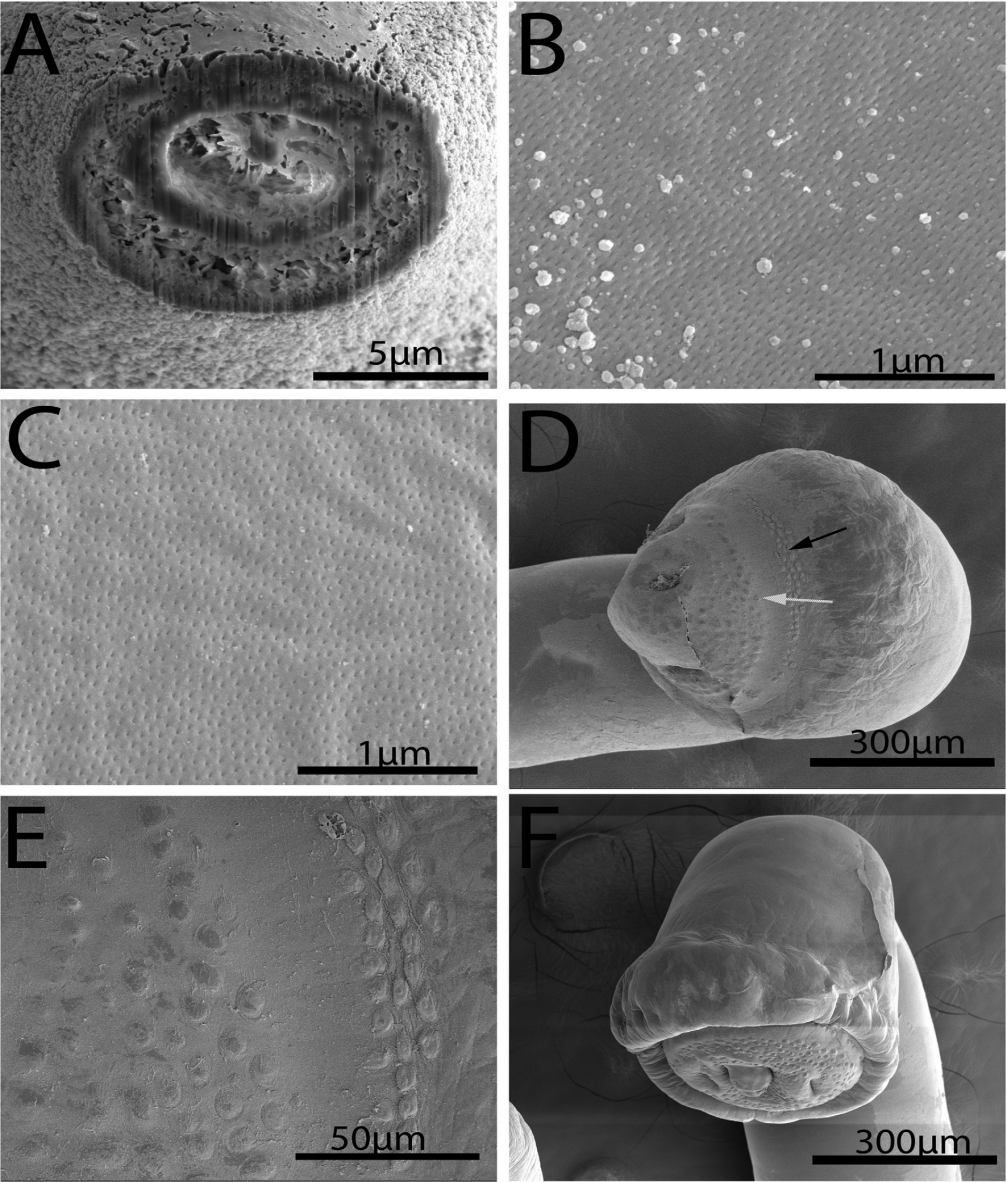
SEM image of specimens of *Rhadinorhynchus oligospinosus* from *Scomber japonicus* and *Trachurus murphyi* from off the Pacific coast of Peru. (A) A gallium cut section of a trunk spine at its base showing the differentiation between its cortical and core layers and associated spaces. (B) Micropores at the anterior part of the trunk. (C) Micropores at the Bmid-section of the trunk. Note the difference in the diameter and distribution of micropores at different trunk regions. (D) A dorsal view of the bursa showing the distribution of the outer zone (black arrow) and the inner zone (white arrow) of sensory receptors. (E) A larger magnification of sensory receptors in the outer and inner zones. (F) A ventro-dorsal perspective of a bursa showing its thick muscular margin and its terminal gonopore and appendage.

*General*: With characters of the genus *Rhadinorhynchus.* Shared structures larger in females than in males. Trunk somewhat small, arched dorsally, uniformally cylindrical, spinose anteriorly in two fields separated by aspinose zone, widest at the region of posterior spines. (Figs. 1A, 1B, 2A). Cuticular surface flat with electron dense micropores of varying diameter and density in different trunk regions (Figs. 3B, Figs. 3C). Trunk spines conical with pointed tips and two internal support rods connected basally (Figs. 1F, 1G). Spines wide and rounded basally (Fig. 2F) with broad fenestrated cortical layer and large separated center core (Fig. 3A). Anterior field of spines in 0–3 irregular circles of 0–2 ventral, 1–3 dorsal, and 8–25 lateral spines in males. Corresponding spines in females in 2–4 irregular circles of 2–4 ventral, 2–4 dorsal, and 14–21 lateral spines. Posterior field of spines in irregular circles of ventral and lateral spines only extending posteriorly up to the level of posterior end of the proboscis receptacle in both males and females. Posterior field of spines of 3–7 ventral and 9–43 lateral spines in males, and 10–15 ventral and 37–45 lateral spines in females. Proboscis long, cylindrical, usually arched (Figs. 2A, 2B) slightly wider anteriorly (Figs. 1A, 1B, 2A, 2B), with 11–14 longitudinal alternating rows of 20–22 hooks each. Hooks smallest anteriorly (Fig. 2C), largest at middle, gradually smaller posteriorly except at basal circle of abruptly larger hooks. Ventral hooks robust with simple, prominent, posteriorly directed roots gradually transitioning to dorsal slender, smaller, hooks with discoid roots (Figs. 1D, 1E). Hooks broadest basally (Fig. 2D) with prominent cortical layer and separated core in cross-sections (Fig. 2E). Neck prominent. Proboscis receptacle markedly longer than proboscis with cephalic ganglion near its middle. Lemnisci digitiform, equal, invariably a little shorter than receptacle. Gonopore terminal in males, subterminal in females, distinctly anterior to bluntly pointed posterior end (Figs. 1A, 1B, 1C).

**Figure 4.**
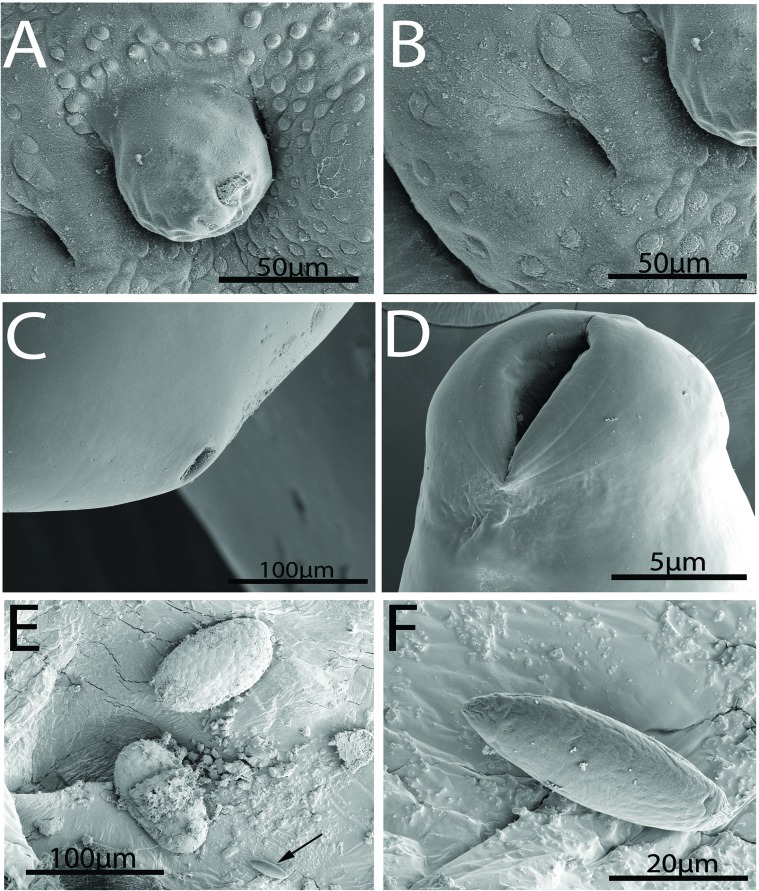
SEM image of specimens of *Rhadinorhynchus oligospinosus* from *Scomber japonicus* and *Trachurus murphyi* from off the Pacific coast of Peru. (A) The terminal genital appendage of a bursa surrounded by inner sensory discs. Note one of the two clefts to left. (B) A larger magnification of one of the two genital clefts with a few sensory discs and part of the terminal genital appendage. (C, D) The various sizes of the female genital orifice are shown with the larger one (D) occupying almost all the trunk diameter at that level; note prominent lips. (E) Two ovarian balls and one mature egg (arrow) from the body cavity of one female specimen. Note comparative sizes. (F) A fully developed egg. Note the lack of any characteristic features or fibrils.

*Male* (based of 11 adults with sperm): Trunk 4.50–11.25 (6.90) mm long by 0.30–0.90 (0.47) mm wide. Anterior trunk spines 52–75 (63) long dorsally, 40–60 (51) long ventrally, and 45–77 (58) long laterally. Larger posterior trunk spines 57–90 (74) long ventrally and 50–87 (68) long laterally. Proboscis 1.49–1.79 (1.65) long by 0.15–0.19 (0.17) wide. Anteriormost hooks smallest, 40–62 (55) long by 7–14 (9) wide at base dorsally and 47–62 (57) long by 7–18 (13) wide at base ventrally. Middle hooks 50–62 (56) by 10–17 (13) dorsally and 65–82 (72) by 18–25 (21) ventrally. Smaller posteriormost hooks 35–50 (44) by 9–13 (11) dorsally and 48–65 (58) by 15–20 (18) ventrally. Longer basal hooks in circle 50–70 (60) by 12–20 (14) dorsally and 62–80 (70) by 15–22 (17) ventrally. Neck 260–364 (312) long dorsally by 156–239 (217) wide at base. Proboscis receptacle 1.90–2.65 (2.30) mm long by 0.18–0.31 (0.23) mm wide. Lemnisci 1.56–2.50 (1.97) mm long by 0.07–0.25 (0.12) mm wide. Reproductive system post-equatorial in compact contiguous structures with genitalia opening into bursa. Anterior testis 468–1875 (852) long by 146–450 (274) wide; slightly larger than posterior testis 489–1952 (807) long by 146–450 (260) wide. Cement glands 4, equal, in two contiguous pairs 291–780 (466) long by 42–166 (108) wide, parallel to ventral common sperm duct and just anterior to Saefftigen’s pouch 333–936 (601) long by 94–229 (159) wide (Fig. 1A). Bursa 551–950 (750) long by 572–825 (698) wide, highly ornate, and complex with thick muscular rim with many sensory papillae in two circular regions and double deep pits around prominent central genital appendage (Figs. 3D–3F, 4A, 4B).

*Female* (based on seven adult females gravid with eggs and some ovarian balls): Trunk 8.02–11.87 (9.93) mm long by 0.42–0.48 (0.43) mm wide. Anterior trunk spines 52–70 (60) long dorsally, 55–65 (62) long ventrally, and 50–72 (60) long laterally. Larger posterior trunk spines 67–90 (85) long ventrally and 72–80 (76) long laterally. Proboscis 1.82–2.31 (2.05) long by 0.23–0.25 (0.24) wide. Middle hooks 52–83 (68) long by 12–27 (18) wide at base dorsally and 75–87 (80) by 20–27 (22) ventrally. Smaller posteriormost hooks 37–75 (56) by 10–17 (13) dorsally and 65–77 (70) by 20–22 (21) ventrally. Longer basal hooks in circle 60–82 (74) by 15–19 (16) dorsally and 70–90 (82) by 17–22 (19) ventrally. Neck 364–395 (385) long dorsally by 224–302 (260) wide at base. Proboscis receptacle 2.81–3.37 (3.08) mm long by 0.23–025 (0.24) mm wide. Lemnisci 2.18–2.76 (2.50) mm long by 0.07–0.11 (0.10) mm wide. Reproductive system 2.70–3.85 (3.16) mm long; 30%–36% (33%) of trunk length, with subventral gonopore (Figs. 1B, 1C). Genital opening and orifice vary considerably in size from very small to almost as wide as posterior trunk diameter (Figs. 4C, 4D). Ovarian balls ovoid with prominently textured surface and eggs oblong (unripe) to fusiform (ripe), bluntly pointed (Figs. 4E, 4F), 62–82 (74) long by 20–27 (24) wide with unremarkable polar prolongation of fertilization membrane (Fig. 1H).

#### Remarks

The specimens of *R. oligospinosus* described in this study are conspecific with those reported in the Peruvian Pacific from *S. japonicus* as *Rhadinorhynchus pristis* Lühe, 1911 by Cruces et al. [8]. This report addressed only prevalence parameters with no taxonomic or descriptive information except for a few SEM images of proboscis and anterior trunk. The SEM images were not of diagnostic quality yet compatible with our observations. Cruces et al. [8] diagnosis confused these specimens with those of *R. pristis* that are, however, large worms (females up to 70.0 mm long) with 14–16 rows of 26 hooks each, weak anterior ventral hooks, only six posterior ventral trunk spines, and large eggs (120 × 20) [4]. At least nine other species of *Rhadinorhynchus* have been misdiagnosed as *R. pristis*; see the following list [2, 4]:

1. *Echinorhynchus pristis* sensu Linton, 1891, in part: synonym of *Rhadinorhynchus ornatus* Van Cleave, 1918.
2. *Echinorhynchus pristis tenuicornis* Linton, 1891 (= *Rhadinorhynchus tenuicornis* Van Cleave, 1947) synonym of *Dollfusentis longispinus* Cable et Linderoth, 1963.
3. *Rhadinorhynchus pristis* sensu Lühe, 1911, in part: synonym of *Rhadinorhynchus lintoni* Cable et Linderoth, 1963.
4. *Rhadinorhynchus pristis* sensu Fukui et Morisita, 1937: synonym of *Rhadinorhynchus seriolae* (Yamaguti, 1963) Golvan, 1969.
5. *Rhadinorhynchus pristis* sensu Chandler, 1943: synonym of *Rhadinorhynchus selkirki* Van Cleave, 1921.
6. *Rhadinorhynchus pristis* sensu Johnston et Edmonds, 1947: synonym of *Rhadinorhynchus johnstoni* Golvan, 1969.
7. *Rhadinorhynchus pristis* sensu Zhukov, 1960: synonym of *Rhadinorhynchus zhukovi* Golvan, 1969.
8. *Rhadinorhynchus pristis* sensu Cable et Linderoth, 1963: synonym of *Rhadinorhynchus dujardini* Golvan, 1969.
9. *Rhadinorhynchus pristis* sensu Solonchenko, 1968 and Kovaleva, 1970 is another species [14].

#### Comparisons

Specimens of *R. oligospinosus* n. sp. are closest to those of *Rhadinorhynchus seriolae* (Yamaguti, 1963) Golvan 1969 [syns. *Nipporhynchus seriolae* Yamaguti, 1963; *Rhadinorhynchus pristis* sensu Fukui and Morisita, 1937] first described from yellowtail (Japanese amberjack), *Seriola quinqueradiata* Temminck and Schlegel (Carangidae), in the prefecture of Mie, Japan [4, 36]. The two species, are, however, separated as follows. In *R. seriolae*, (1) the females are considerably longer (15.0–30.0 mm long) than females of *R. oligospinosus* (8.0–11.9 mm), (2) the proboscis has 12–13 rows of more hooks (23–25) per row each compared to 20–22 hooks per row in our specimens, (3) more ventral posterior trunk spines (11 in males, 20 in females) compared to 3–7 and 10–15 in *R. oligospinosus*, (4) the posterior trunk spines extended well beyond the level of the posterior end of the proboscis receptacle in female specimens of *R. seriolae* but not in *R. oligospinosus*, (5) the lemnisci are markedly shorter than the receptacle in our specimens but slightly longer in Smales’s [36] specimens of *R. seriolae*, (6) in *R. seriolae*, the testes are in the mid-third of the trunk but at the posterior end in *R. oligospinosus*, (7) the elaborate organization of the bursa and the extensive presence of sensory structures in our specimens have not been reported in *R. seriolae* or any other species of *Rhadinorhynchus* by other observers including Fukui and Morisita [11] or Golvan [13]. Kanda’s [19] *Rhadinorhynchus japonicus* Fujita, 1920 interpreted by Ichihara et al. [18] as *R. seriolae* and Ichihara et al.’s [18] own “*R. seriolae*” are of questionable identity as their proboscis had 16–18 rows of 20–26 hooks each. They [18] interpreted forms with such differences in hook counts, among other features, as “varieties” of *R. seriolae*. The validity of *R. japonicus* has, however, been established [4]. (8) *R. seriolae* and *R. oligospinosus* n. sp. have been reported from mackerels (Carangidae and Scombridae). The first species was found in Japan [11, 18, 40] and east Australia [36] but our new species is reported in the Peruvian Pacific (this paper).

#### X-ray scans

**Figure 5.**
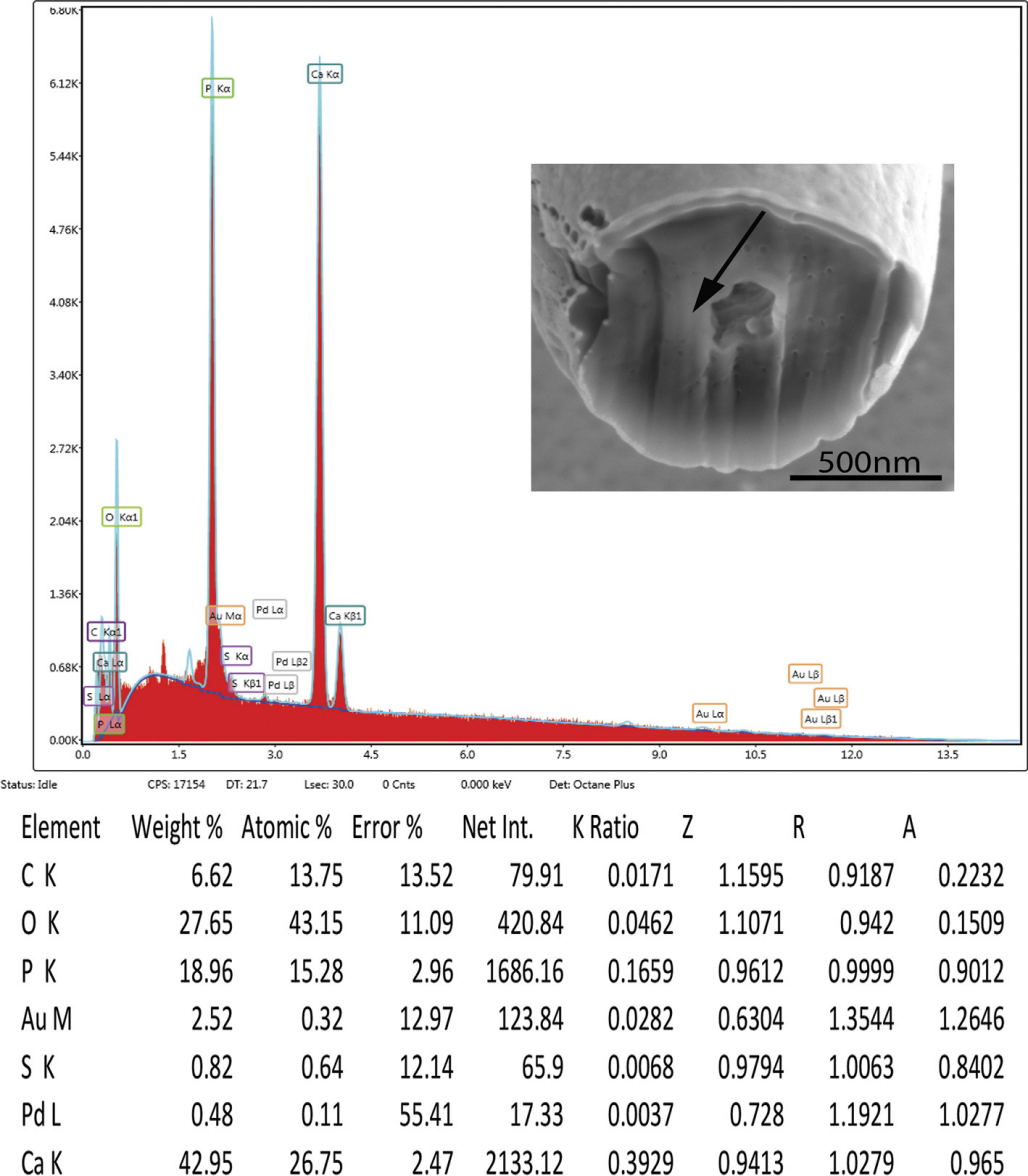
EDAX data for the scan of the center of a cut hook (arrow).

**Figure 6.**
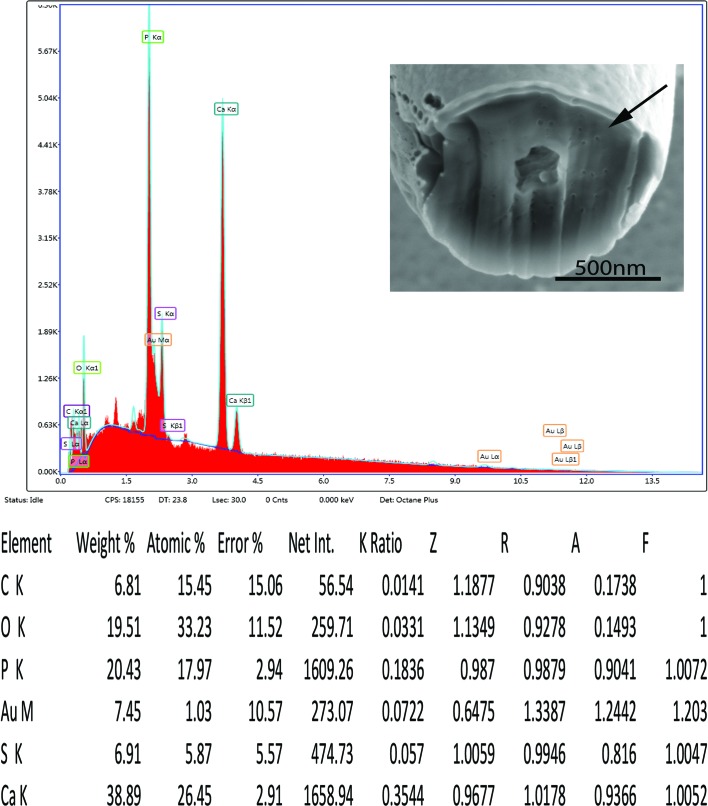
EDAX data for the scan of the edge of a cut hook (arrow).

The X-ray scans (EDAX) of gallium cut hooks are listed in Table 1 and Figures 5 and 6. There is a high content of phosphorus and calcium in the middle and edge scans with a varying amount of sulfur. Other common elements of living organisms are also recorded (carbon and oxygen) as well as elements used for specimen preparation (gallium, palladium, gold). Sulfur has a differential higher concentration at the edge (6.91 wt.%) than the middle of cut hooks (0.82 wt.%). This element is part of the prominent outer layer of most acanthocephalan hooks and is a major contributor to the hardening process of this attachment structure. Our results are comparable to those of mammalian teeth enamel. A similar EDAX study was carried out on the proboscis hooks of *Echinorhynchus baeri* Kostylew, 1928 showing that the large hooks have higher calcium, phosphorus, and sulfur than miniature rootless hooks [5]. Additional studies [15, 16, 37] have reported on hardened hooks and teeth enamel using elemental analysis (XEDs, EDAX).

**Table 1.** X-Ray data for the middle and edge scans from the gallium cut proboscis hooks of specimens of *Rhadinorhynchus oligospinosus* showing *c*enter and edge of the mid cut.

Element	Center cut		Edge cut	
	Weight %	Atomic %	Weight %	Atomic %
Phosphorous (P)*	18.96	15.28	20.43	17.97
Sulfur (S)	0.82	0.64	6.91	5.87
Calcium (Ca)	42.95	26.75	38.89	26.45

* The other listed elements; Carbon (C) and Oxygen (O) are common in all living animals; Gold (Au) and Palladium (Pd) are used to coat the specimens, usually an 18 nm layer. Gallium (Ga) is used to cut the specimen.

## Discussion

Amin et al. [4] described two new species of *Rhadinorhynchus*, listed 36 others, and provided keys to all 38 valid species. Only four more species of *Rhadinorhynchus* were described since, from marine fishes off Australia [36]. The 42 valid species of *Rhadinorhynchus* and *Rhadinorhynchus oligospinosus* n. sp. include 20 species from the Pacific Ocean especially off Australia, Japan, and Vietnam. These species are:

1. *Rhadinorhynchus bicircumspinus* Hooper, 1983 from New South Wales, Australia.
2. *Rhadinorhynchus biformis* Smales 2014. Heron Island, Australia.
3. *Rhadinorhynchus carangis* Yamaguti, 1939 from Japanese Inland Sea, Japan.
4. *Rhadinorhynchus chongmingnensis* Huang, Zheng, Deng, Fan et Ni, 1988 from Chongming, China.
5. *Rhadinorhynchus cololabis* Laurs et McCauley, 1964 from Oregon, USA.
6. *Rhadinorhynchus decapteri* Parukhin et Kovalenko, 1976 from Hawaii, USA.
7. *Rhadinorhynchus ditrematis* Yamaguti, 1939 from Japanese Inland Sea, Japan.
8. *Rhadinorhynchus dorsoventrospinosus* Amin, Heckmann, Ha 2011 from Halong Bay, Vietnam.
9. *Rhadinorhynchus johnstoni* Golvan, 1969 from South Australia.
10. *Rhadinorhynchus laterospinosus* Amin, Heckmann, Ha, 2011 from Halong Bay, Vietnam.
11. *Rhadinorhynchus oligospinosus* n. sp. from Port of Chicama, La Libertad, Peru.
12. *Rhadinorhynchus ornatus* Van Cleave, 1918 from the Atlantic coast of the USA, Japan, and the Pacific Ocean off South America.
13. *Rhadinorhynchus pichelinae* Smales, 2014 from Point Peron, West Australia.
14. *Rhadinorhynchus polydactyli* Smales, 2014 from Moreton Bay, Queensland, Australia.
15. *Rhadinorhynchus polynemi* Gupta and Lata, 1967 from India and NE Australia.
16. *Rhadinorhynchus pomatomi* Smales, 2014. New Brighton, New South Wales, Australia.
17. *Rhadinorhynchus selkirki* Van Cleave, 1920 from Juan Fernandez Island, Chili.
18. *Rhadinorhynchus seriolae* (Yamaguti, 1963) Golvan, 1969 from Japan and Australia.
19. *Rhadinorhynchus trachuri* Harada, 1935 from a Tokyo market, Japan.
20. *Rhadinorhynchus zhukovi* Golvan, 1969 from the Kuril Islands, Japan-Russia.

*Rhadinorhynchus oligospinosus* n. sp. is distinguished from the above 19 species as well as from the remaining species of the genus by the following combination of characters: proboscis with 11–14 rows of 20–22 hooks each with basal hooks in a perfect ring, trunk with two fields of spines separated by aspinose zone, anterior trunk spines in a few complete circles, and posterior trunk spines only ventral and lateral extending posteriorly only to level posterior end of proboscis receptacle in both males and females.

## Conclusions

*Rhadinorhynchus oligospinosus* n. sp. is the only species of the currently recognized 43 species of the genus *Rhadinorhynchus* that is found off the coast of Peru. Another 19 species known in the Pacific Ocean are mostly found off the coasts of Australia, Japan, and Vietnam. Most of the remaining species are found off the coasts of India, Africa, and the Arabian Sea. The closest species to *R. oligospinosus* is *R. seriolae* from Japan and Australia which is, however, distinguished from our species by the distribution of posterior trunk spines and proboscis armor, among other features. *Rhadinorhynchus ornatus*, a more distant species from *R. oligospinosus*, similarly occupies a long stretch of the Pacific off the South American coast but mostly along Colombia and Ecuador but with extended distribution from species of tuna in Atlantic North America and the Sea of Japan [3]. The key by Amin et al. [4] conveniently separates those species, and others. In X-ray scans of gallium-cut hooks, the core and edge of hooks were shown to have a high content of phosphorus and calcium but hook edge had a markedly higher concentration of sulfur. Sulfur is a major contributor of the hardening process of acanthocephalan hooks as has previously been demonstrated in other acanthocephalan species.

## Conflict of interest

The authors declare that they have no conflict of interest.
